# Supplementary material for: COZOID: contact zone identifier for visual analysis of protein-protein interactions
Source: BMC Bioinformatics. 2018 Apr 6;19:125. doi: 10.1186/s12859-018-2113-6 (PMC5889581; doi:10.1186/s12859-018-2113-6)
Supplement: Supplementary file 2 — Software build. Executable binary file of the software tool. (ZIP 118,784 kb) [file 12859_2018_2113_MOESM2_ESM.zip › cozoid/java/docs/beansbinding-1.2.1-doc.zip!/org/jdesktop/el/impl/lang/ExpressionBuilder.html]

ExpressionBuilder (Beans Binding)


---


|  |  |  |
| --- | --- | --- |
| **Overview** \| **Package** \| **Class** \| **Use** \| **Tree** \| **Deprecated** \| **Index** \| **Help** | |  |
| **PREV CLASS**   **NEXT CLASS** | **FRAMES**    **NO FRAMES**     **All Classes** |  |
| SUMMARY: NESTED \| FIELD \| CONSTR \| METHOD | DETAIL: FIELD \| CONSTR \| METHOD |  |


---


## org.jdesktop.el.impl.lang Class ExpressionBuilder

```
java.lang.Object
  org.jdesktop.el.impl.lang.ExpressionBuilder
```

**All Implemented Interfaces:**: NodeVisitor

---

``` public final class ExpressionBuilder extends java.lang.Object implements NodeVisitor ```

---

**Constructor Summary**

`ExpressionBuilder(java.lang.String expression,
ELContext ctx)`


| **Method Summary** | |
| --- | --- |
| `MethodExpression` | `createMethodExpression(java.lang.Class expectedReturnType, java.lang.Class[] expectedParamTypes)` |
| `static Node` | `createNode(java.lang.String expr)` |
| `ValueExpression` | `createValueExpression(java.lang.Class expectedType)` |
| `void` | `visit(Node node)` |

**Methods inherited from class java.lang.Object**

`clone, equals, finalize, getClass, hashCode, notify, notifyAll, toString, wait, wait, wait`

**Constructor Detail**

### ExpressionBuilder

```
public ExpressionBuilder(java.lang.String expression,
                         ELContext ctx)
                  throws ELException
```

**Throws:**: `ELException`


**Method Detail**

### createNode

```
public static final Node createNode(java.lang.String expr)
                             throws ELException
```

:   **Throws:**: `ELException`

---


### visit

```
public void visit(Node node)
           throws ELException
```

:   **Specified by:**: `visit` in interface `NodeVisitor`
:   **Throws:**: `ELException`

---


### createValueExpression

```
public ValueExpression createValueExpression(java.lang.Class expectedType)
                                      throws ELException
```

:   **Throws:**: `ELException`

---


### createMethodExpression

```
public MethodExpression createMethodExpression(java.lang.Class expectedReturnType,
                                               java.lang.Class[] expectedParamTypes)
                                        throws ELException
```

:   **Throws:**: `ELException`


---


|  |  |  |
| --- | --- | --- |
| **Overview** \| **Package** \| **Class** \| **Use** \| **Tree** \| **Deprecated** \| **Index** \| **Help** | |  |
| **PREV CLASS**   **NEXT CLASS** | **FRAMES**    **NO FRAMES**     **All Classes** |  |
| SUMMARY: NESTED \| FIELD \| CONSTR \| METHOD | DETAIL: FIELD \| CONSTR \| METHOD |  |


---
